# Chemical-free and scalable process for the fabrication of a uniform array of liquid-gated CNTFET, evaluated by KCl electrolyte

**DOI:** 10.1038/s41598-021-83451-2

**Published:** 2021-02-17

**Authors:** Pankaj B. Agarwal, Navneet Kumar Thakur, Rishi Sharma, Parul Singh, Joshy Joseph, Chaturvedula Tripura

**Affiliations:** 1 Smart Sensors Area, CSIR-Central Electronics Engineering Research Institute (CSIR-CEERI), Pilani, Rajasthan 333031 India; 2 Academy for Scientific and Innovative Research (AcSIR), Ghaziabad, Uttar Pradesh 201002 India; 3 Chemical Sciences and Technology Division, CSIR-National Institute for Interdisciplinary Science and Technology (CSIR-NIIST), Thiruvananthapuram, 695019 India; 4 CCMB-Annexe-II, Medical Biotechnology Complex, CSIR-Centre for Cellular and Molecular Biology (CSIR-CCMB), Uppal Road, Uppal, Hyderabad, Telangana 500039 India

**Keywords:** Engineering, Nanoscience and technology

## Abstract

Biosensors based on liquid-gated carbon nanotubes field-effect transistors (LG-CNTFETs) have attracted considerable attention, as they offer high sensitivity and selectivity; quick response and label-free detection. However, their practical applications are limited due to the numerous fabrication challenges including resist-based lithography, in which after the lithography process, the resist leaves trace level contaminations over the CNTs that affect the performance of the fabricated biosensors. Here, we report the realization of LG-CNTFET devices using silicon shadow mask-based chemical-free lithography process on a 3-in. silicon wafer, yielding 21 sensor chips. Each sensor chip consists of 3 × 3 array of LG-CNTFET devices. Field emission scanning electron microscope (FESEM) and Raman mapping confirm the isolation of devices within the array chip having 9 individual devices. A reference electrode (Ag/AgCl) is used to demonstrate the uniformity of sensing performances among the fabricated LG-CNTFET devices in an array using different KCl molar solutions. The average threshold voltage (V_th_) for all 9 devices varies from 0.46 to 0.19 V for 0.1 mM to 1 M KCl concentration range. This developed chemical-free process of LG-CNTFET array fabrication is simple, inexpensive, rapid having a commercial scope and thus opens a new realm of scalable realization of various biosensors.

## Introduction

Carbon nanotubes (CNTs) have attracted most attention due to the unique combination of their electrical, optical, mechanical and thermal properties^1,2^, which make them suitable for incorporation in platforms such as chemiresistors^3,4^, field-effect transistors (FETs)^5,6^, supercapacitors^7^, and other biosensing devices^8^. For comprehensive use of the biosensing capabilities of CNTs, the approach of their integration into different electrical platforms is one of the important aspects. As-synthesized single-walled CNTs (SWNTs) consist of a mixture of semiconducting (s-) and metallic (m-) nanotubes. The use of this mixture will yield to the variations in device-to-device performance and particularly m-SWNTs degrade the performance of the electronic devices. The s-SWNTs are preferred for thin-film based sensor platforms due to its outstanding charge transport quality and small size (compatible to biomolecules), which make them extremely sensitive to small changes in the surroundings whether it is a gaseous or a liquid environment^9,10^. The behaviour of SWNTs-based FET devices for chemical/biochemical sensing have been explored with single SWNT^11^ and multiple SWNTs random network^12,13^ between source-drain electrodes, integrated by growth or by deposition techniques with back-gated^14^, top-gated^11^ biasing, and with or without liquid environment. The deposition of SWNTs random network for the fabrication of the individual device is simple and suitable; however, it is challenging to develop the process for realizing the uniform array of devices, reproducible for commercial production^13^.

For the fabrication of a uniform array of devices, there are many solution process-based ex situ CNTs deposition techniques which mainly includes inkjet printing^15,16^, photolithographic patterning^17,18^, drop-casting^19^, dip-coating^20^, electrophoretic deposition^21^, spin-coating^22^, and spray-coating^3,23^. Low-cost inkjet printers have lower resolution, control of drop size and spacing between the line features; whereas commercial inkjet printers are expensive^24^. Sometimes, few additional steps such as substrate modification^15^, dispersant removal^24^ etc. are also required in inkjet printing technique, which results in poor electrical conductivity of the CNTs as well as increased device fabrication cost. In the case of CNTs-based sensing platforms, fabricated by a photolithography process, it has been observed that after photoresist removal, their traces are the sources for signal noise which results in the deterioration in their electrical performance^25^. Apart from this, electrophoretic deposition techniques of the CNTs have adhesion issues on semiconducting and metal surfaces^21^. The spin-coating process is not suitable in case of large-size substrates, due to the difficulty in spinning them at high speed, which results in large thickness variation in CNTs film^26^. In the drop-casting method also, arbitrarily thick films of CNTs results in degradation of FET electrostatic control^27,28^. From a commercial point-of-view, though the spray-coating process can deposit uniform layers, nevertheless further investigations are required specifically to use this technique to pattern CNTs with large area uniformity over various kinds of solid/flexible substrates such as silicon, glass, polydimethylsiloxane (PDMS), poly(methyl methacrylate) (PMMA), polyether ether ketone (PEEK) etc^29^.

In this paper, we report direct patterning of a uniform thin film of s-SWNTs using chemical-free shadow mask lithography instead of conventional photolithography. A silicon shadow mask with openings is fabricated using silicon bulk-micromachining and then successfully aligned with the predefined source-drain electrode structures followed by optimized spray coating process to fabricate an array of LG-CNTFET devices. This process is chemical-free, economic, rapid, ease in use and scalable for commercial production of LG-CNTFET device array as a chemical/biochemical sensing platform. The measured resistances between source and drain assure uniformity among the fabricated array of devices. The sensing performance of the fabricated LG-CNTFET biosensing platform with 0.1 mM to 1 M range of KCl concentrations confirms the consistency and uniformity among the complete array of devices.

## Materials and methods

### Materials

Tetramethylammonium hydroxide (TMAH) (25% solution in water) was purchased from Merck. 1,2-Dichlorobenzene (DCB) (Anhydrous, 99%,) and reference electrode (Ag/AgCl) were purchased from Sigma-Aldrich. Pristine s-SWNTs (IsoNanotubes-S) was purchased from Nanointegris. Sylgard 184 silicone elastomer was purchased from Dow Corning.

### Silicon shadow mask fabrication

**Figure 1:**
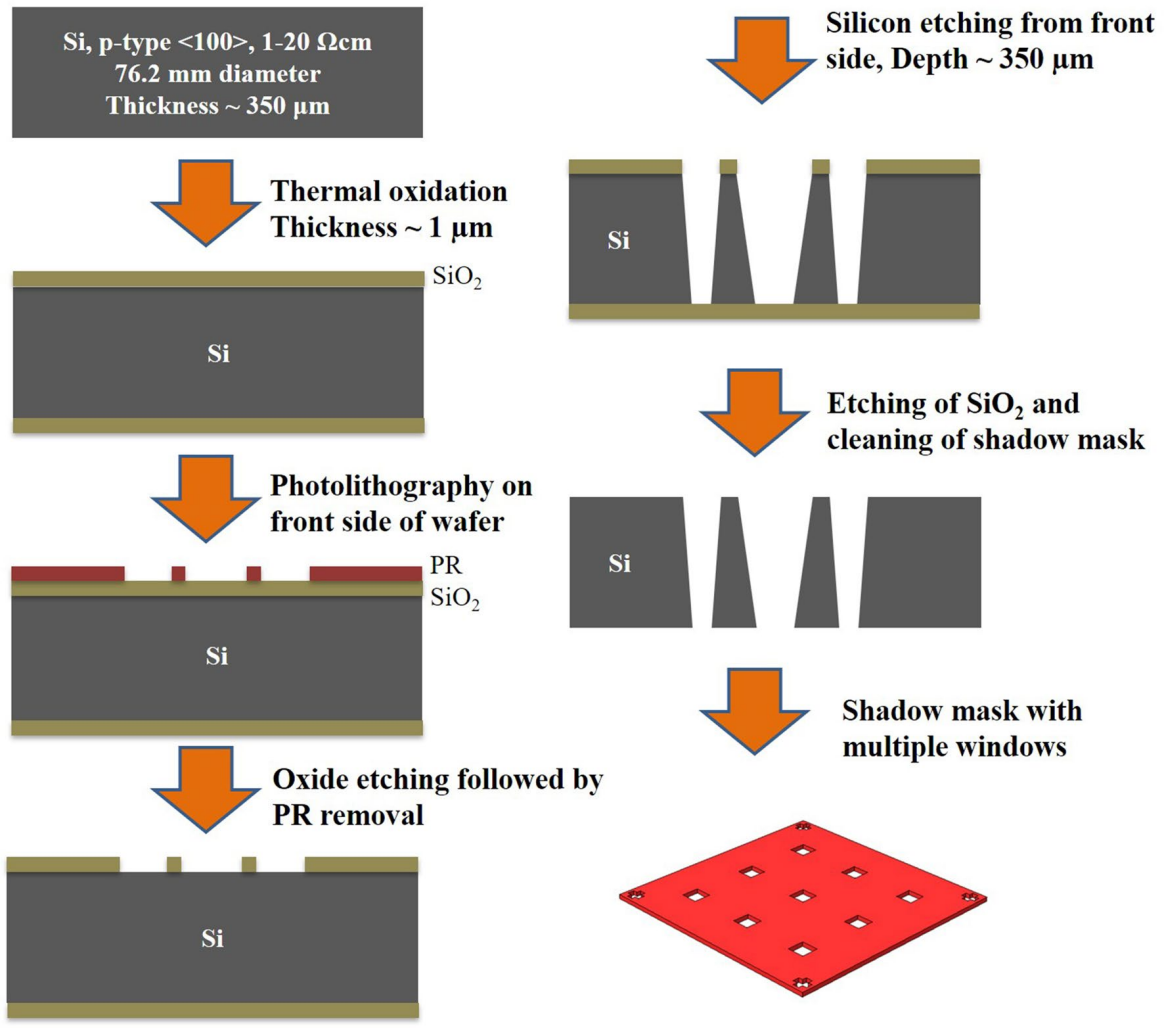
Schematic of a silicon shadow mask showing the windows, which are used to fabricate 3 × 3 array of devices over a single chip.

The method of silicon shadow mask fabrication was adopted with minor modifications from our earlier work as shown in Fig. 1^30^. First silicon dioxide (SiO_2_) of ~ 1 μm thickness was grown over 350 μm thick silicon wafer using thermal oxidation and windows of 400 μm × 400 μm size were patterned using photolithography. TMAH solution was used for bulk micromachining of silicon at 80 °C, having an etch rate of ∼ 24 μm/h. For a single LG-CNTFET chip, 9 windows were opened in a silicon shadow mask to realize an array of 3 × 3 devices.

### Spray coating optimization

In-house developed spray coating set-up was used to prepare a thin film of s-SWNTs over 3-in. (diameter) silicon wafer^30^. The setup comprises of ultrasonic atomizer probe, motorized X–Y stage, a hot plate for in situ solvent evaporation, syringe pump for precise flow control of s-SWNTs suspension, and an exhaust fan. The suspension was prepared using pristine s-SWNTs in DCB with a concentration of 0.1 mg/ml^31^. Uniform s-SWNTs thin film was achieved first on bare silicon substrates through optimization of spray coating parameters namely substrate temperature, substrate-spray nozzle distance, solution concentration, and flow rate. A four-probe sheet resistance measurement (QuadPro, Signatone) was used to map the sheet resistance of these s-SWNTs coated bare silicon substrates. The optimized value of uniform sheet resistance was ~ 0.9 to 1.4 kΩ/square.

### LG-CNTFET array fabrication

**Figure 2:**
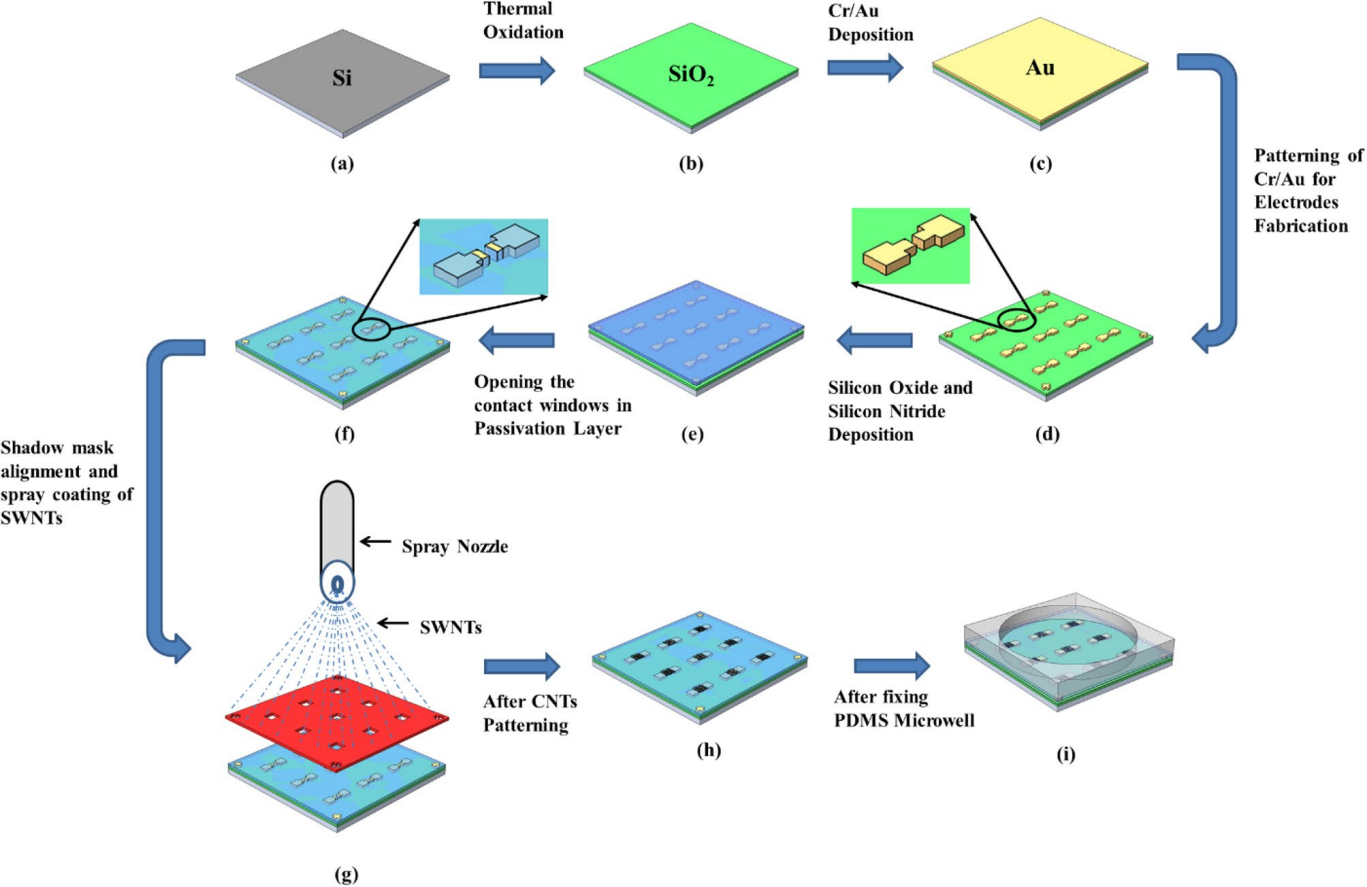
Schematic diagram, showing the fabrication of 3 × 3 array of LG-CNTFETs.

**Figure 3:**
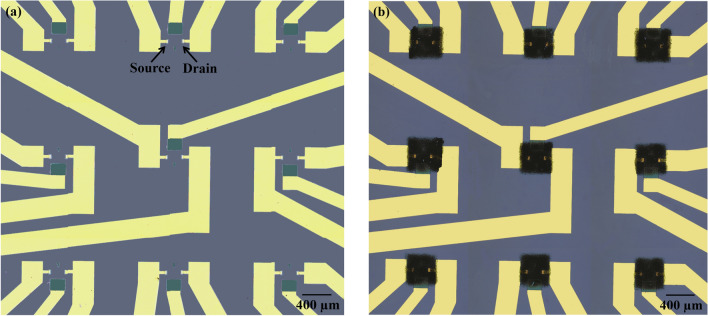
The snapshots of diced s-SWNTs chip with source-drain electrodes in 3 × 3 array of devices, (**a**) before and, (**b**) after s-SWNTs spray.

To fabricate the array of devices, silicon wafer <100> , p-type, 3-in. diameter with resistivity 1–20 Ω-cm was RCA cleaned and (Fig. 2a) and thermal oxide was grown at 1100 °C to achieve silicon dioxide of 1 µm thickness (Fig. 2b). The metal contacts were patterned via photolithography of Cr/Au (300 Å/2500 Å), which were deposited using DC sputtering (Fig. 2c). After wet etching of Cr/Au (Fig. 2d), plasma-enhanced chemical vapour deposition (PECVD) was used to deposit oxide/nitride layers of thickness 1000 Å/4000 Å for passivation purpose (Fig. 2e). Second photolithography was carried out to open only source-drain windows and contact pads in the passivation layer for connections with the s-SWNTs network (Fig. 2f). The patterned substrate with an array of source-drain electrodes pairs was ready for introducing the s-SWNTs as channel elements. This wafer was first aligned with the fabricated reusable silicon shadow mask with the help of in-house developed shadow mask aligner^32^. After alignment, the assembly was ready for spray coating process (Fig. 2g). As discussed in the previous section, the optimized s-SWNTs spray coating process was adopted to fabricate the actual device array over the wafer, of which temperature was maintained ~ 180 °C. This wafer has 189 devices and distributed over 21 individual sensor chips. After spray coating, the device wafer was separated from the shadow mask and diced into 21 chips of 12.7 mm × 12.7 mm size. The individual chip consists of 3 × 3 array of devices (Fig. 2h) followed by fixing the PDMS well (Fig. 2i). The individual chip with source-drain electrodes of an actual fabricated array of s-SWNTs devices before and after s-SWNTs spray is shown in Fig. 3a,b, respectively. The third electrode corresponding to each device (Fig. 3a) is designed for exploring the possibility of integration of on-chip Ag/AgCl electrodes in future.

**Figure 4:**
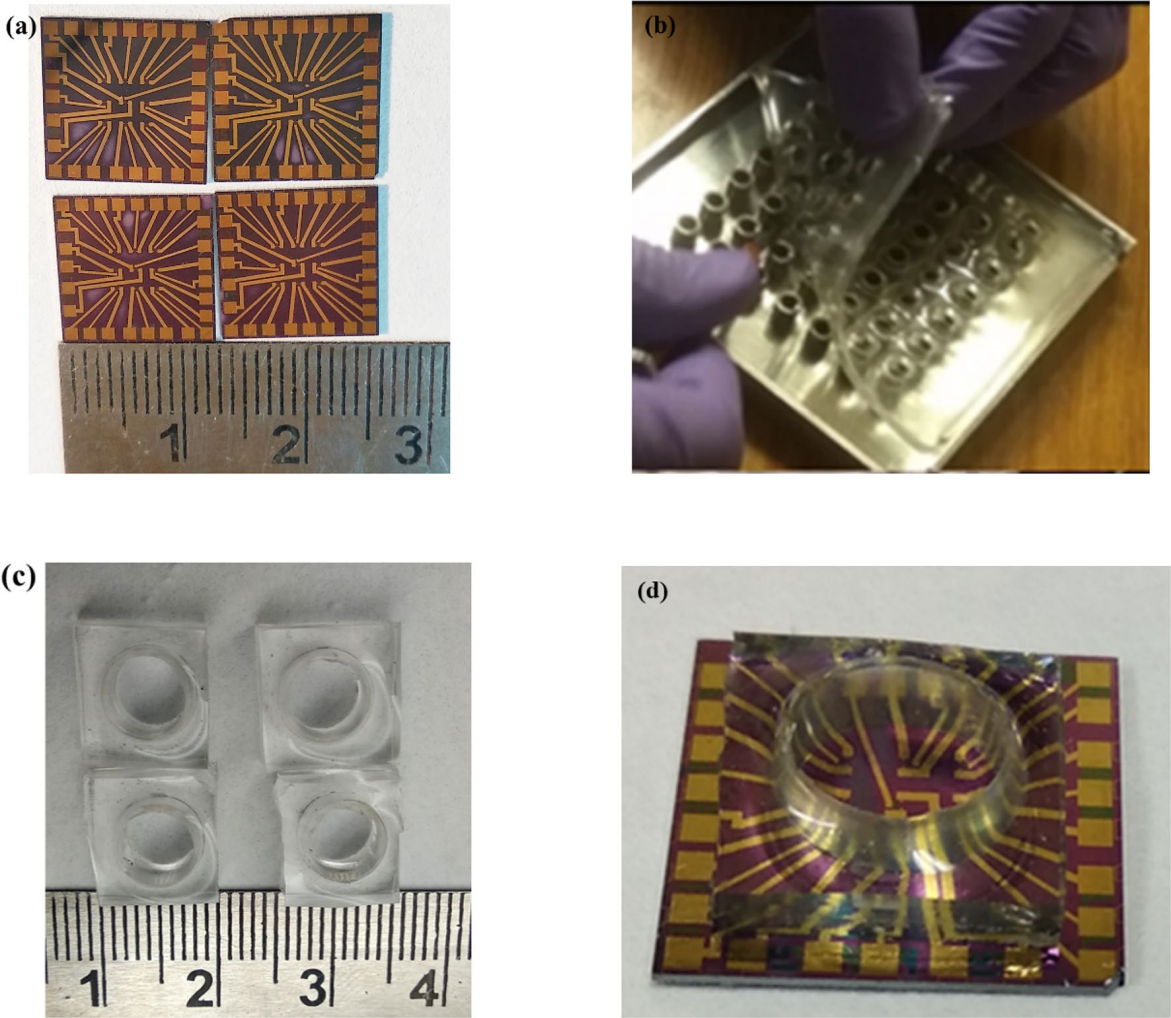
(**a**) Photos of individual chips after dicing, (**b**) peeling off the cured well array from SS stencil (**c**) individual well after dicing, and (**d**) fixing of PDMS well over the fabricated chip.

After dicing the wafer, the snapshot of an individual chip is shown in Fig. 4a. To accommodate and confine different testing fluids over the devices; PDMS wells of 6 mm diameter and 15 mm height were fabricated using Sylgard 184 silicone elastomer base and curing agent in the ratio of 10:1^33^. The PDMS mixture was stirred manually and poured in stainless steel (SS) mould (Fig. 4b). It is then kept in a vacuum desiccator to remove trapped air bubbles. The mixture was cured at 80 °C for 30 min followed by separation of wells by dicing using SS blade. The individual wells (Fig. 4c) were treated with oxygen plasma for 30 s followed by their fixing over the chip (Fig. 4d).

## Results and discussion

### Device uniformity

**Figure 5:**
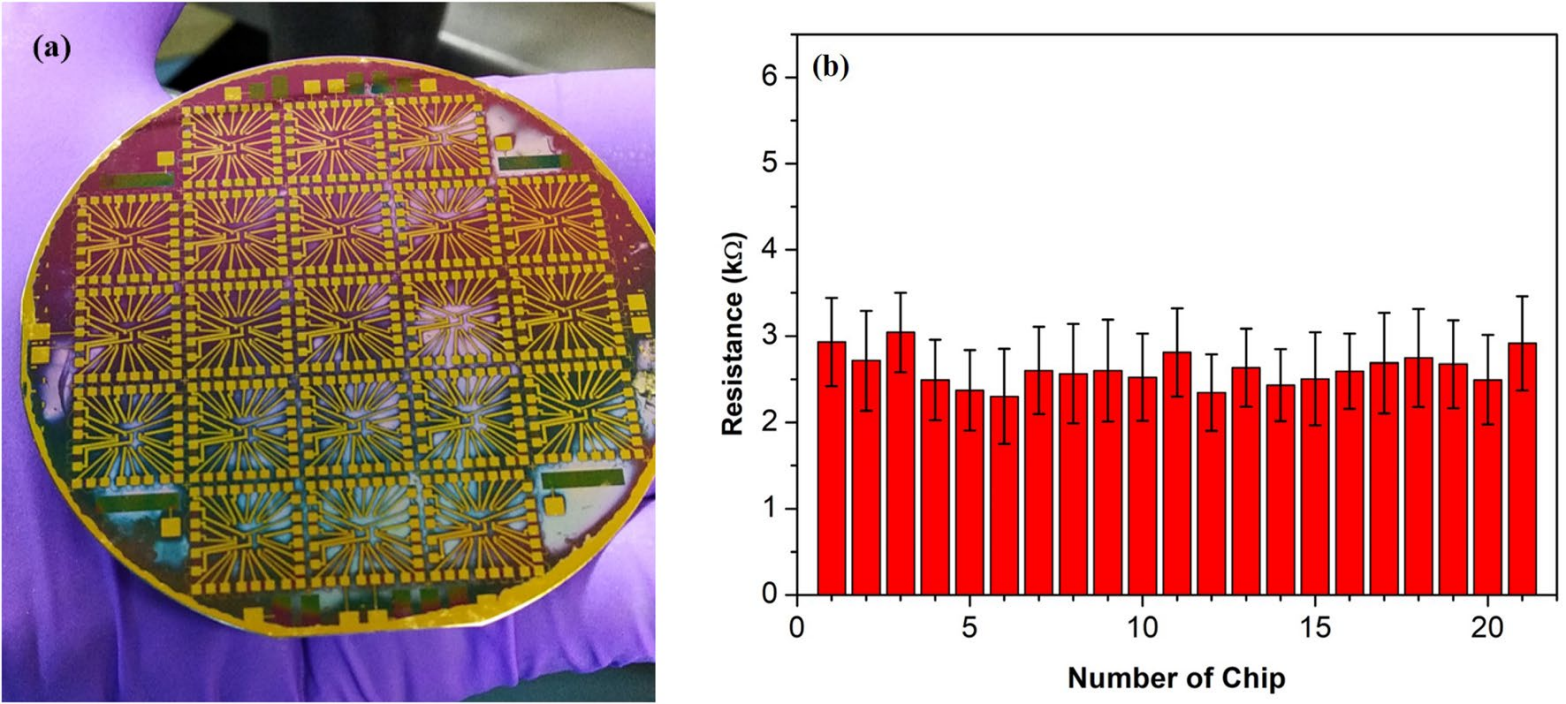
(**a**) Photograph of a 3-in. silicon wafer with 21 chips of LG-CNTFET array, and (**b**) bar-chart of average resistance of individual chips of the fabricated devices with the deposited s-SWNTs network, shows the uniformity among 21 chips of the 3-in. wafer.

First, the resistances of all 189 devices in 21 chips have been measured to assess the uniformity of spray-coated s-SWNTs on the 3-in. substrate. Figure 5a shows the photograph of a complete 3-in. wafer with multiple sensor chips. The single-chip consists of an array of 3 × 3 devices, in which the distance between two adjacent s-SWNTs spray portions of the individual devices is ~ 1.6 mm. Figure 5b, shows the bar-chart of 21 array chips having an average value of source-drain resistances nearly 3 kΩ, which confirms the uniformity across the wafer. One of these chips was chosen for all further electrical measurements by using different KCl molar concentrations. This chip has the majority of devices (7 out of 9 devices), with source-drain resistance in the range of 1–3 kΩ, while remaining two devices has resistances 3.5 kΩ and 6.9 kΩ, respectively.

To confirm the isolation among the fabricated devices, the resistances were measured between various pads and found open circuit except for the resistance between the two pads of the corresponding device in an array.

### Raman and FESEM characterization

**Figure 6:**
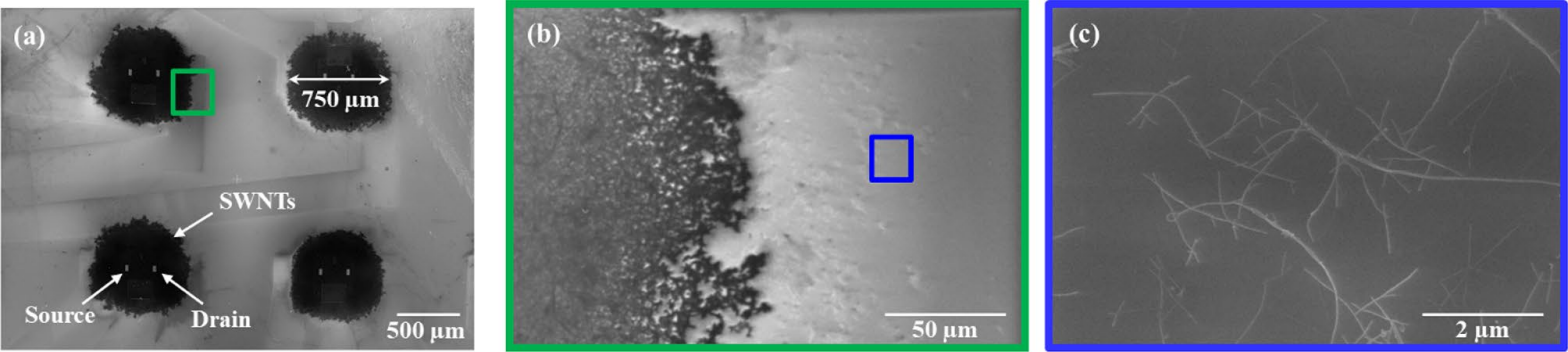
FESEM images (**a**) show the confined network of s-SWNTs between the electrodes ( source-drain) for individual devices (**b**) the enlarged portion (green box) in (**a**) shows the blurring effect, and (**c**) the enlarged portion (blue box) in (**b**) i.e. the outermost portion of the single device confirms the discontinuity in s-SWNTs film.

The spray-coated chip was characterized using FESEM (Quanta FEG 250, FEI) and Raman (InVia, Renishaw) to confirm the presence of s-SWNTs network between the source-drain as well as to ensure isolation of devices in a chip. FESEM image (Fig. 6a) shows the isotropic spray of s-SWNTs over the prefabricated source-drain structure in a circular area of ~ 750 μm diameter larger than the shadow mask opening i.e. 400 μm. There is always a gap between the shadow mask and the source-drain substrate due to inherent bow in the wafers, the s-SWNTs deposited through windows diffuse beyond the shadow mask opening area (Fig. 3b) and there is a progressive reduction of the thickness of deposited s-SWNTs (Fig. 6b), and eventually, their complete closure, as shown in Fig. 6c^34^. The zoomed areas in Fig. 6b and further in 6c confirms, how the s-SWNTs density decreases as we move away from the spray-coated zone of a single device in a chip, and this separation helps to isolate the devices from each other.

**Figure 7:**
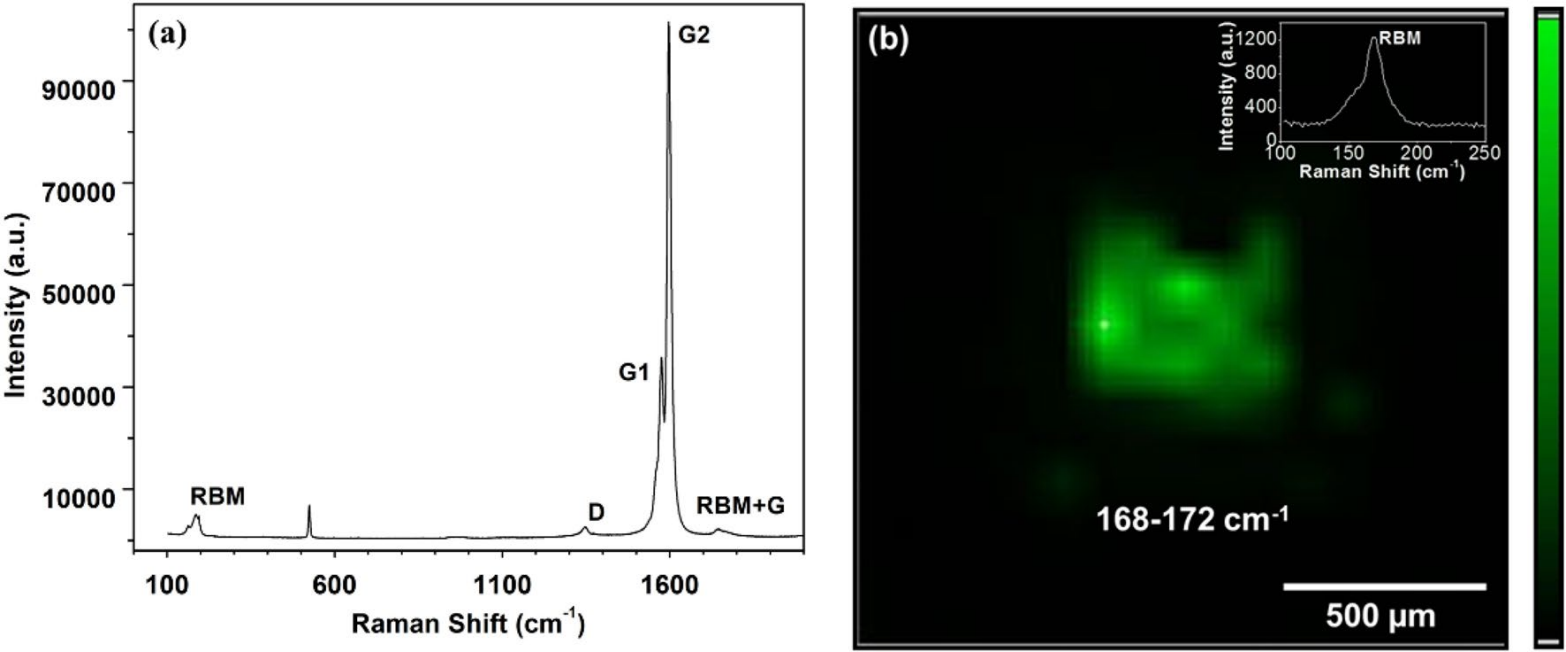
Raman (**a**) spectra of s-SWNTs and (**b**) mapping of the area between source-drain electrodes show the presence of s-SWNTs in the confined area of a single device.

Raman spectra of the channel region were acquired using 532 nm laser with 1 mW power as shown in Fig. 7a. Radial breathing mode (RBM) peak at 169 cm^−1^ confirms the presence of SWNTs, while two distinguishable peaks, G1 at 1575 cm^−1^ and G2 at 1589 cm^−1^, indicates that the SWNTs are semiconducting^35^. To confirm the isolation between the adjacent sprayed s-SWNTs region, the Raman mapping was carried out for a single device of an array using pointwise data collection with the spacing of 20 µm in 1.6 mm × 1.6 mm area (Fig. 7b). In the mapping spectra the contour corresponding to RBM peak range 168–172 cm^−1^, also ensures confinement of sprayed s-SWNTs patterns, therefore it will not interfere with the nearby devices/sensors.

### Electrical measurements

Fabricated LG-CNTFETs array platform was electrically tested using 2-channel source-measure unit (SMU) (B2902A, Keysight) with different KCl solution of the concentrations ranging from 0.1 mM to 1 M. For each electrical measurement, 300 µl KCl solution of required molarity was filled in the capped PDMS well over the device and Ag/AgCl reference electrodes, dipped in the filled KCl solution were used as top-gate for FET measurements^36^.

The output characteristics for the individual device were acquired with drain-source voltage (V_ds_) of 0–0.2 V at different gate voltages (V_gs_) ranging from − 0.1 to 0 V and transfer characteristics were taken for V_gs_ of − 0.4 to 0.8 V at constant V_ds_ bias of 0.1 V. After each experiment with varying the molar concentration of KCl solution, the chip was thoroughly cleaned with deionized (DI) water and dried with the nitrogen (N_2_) for the next set of experiments.

**Figure 8:**
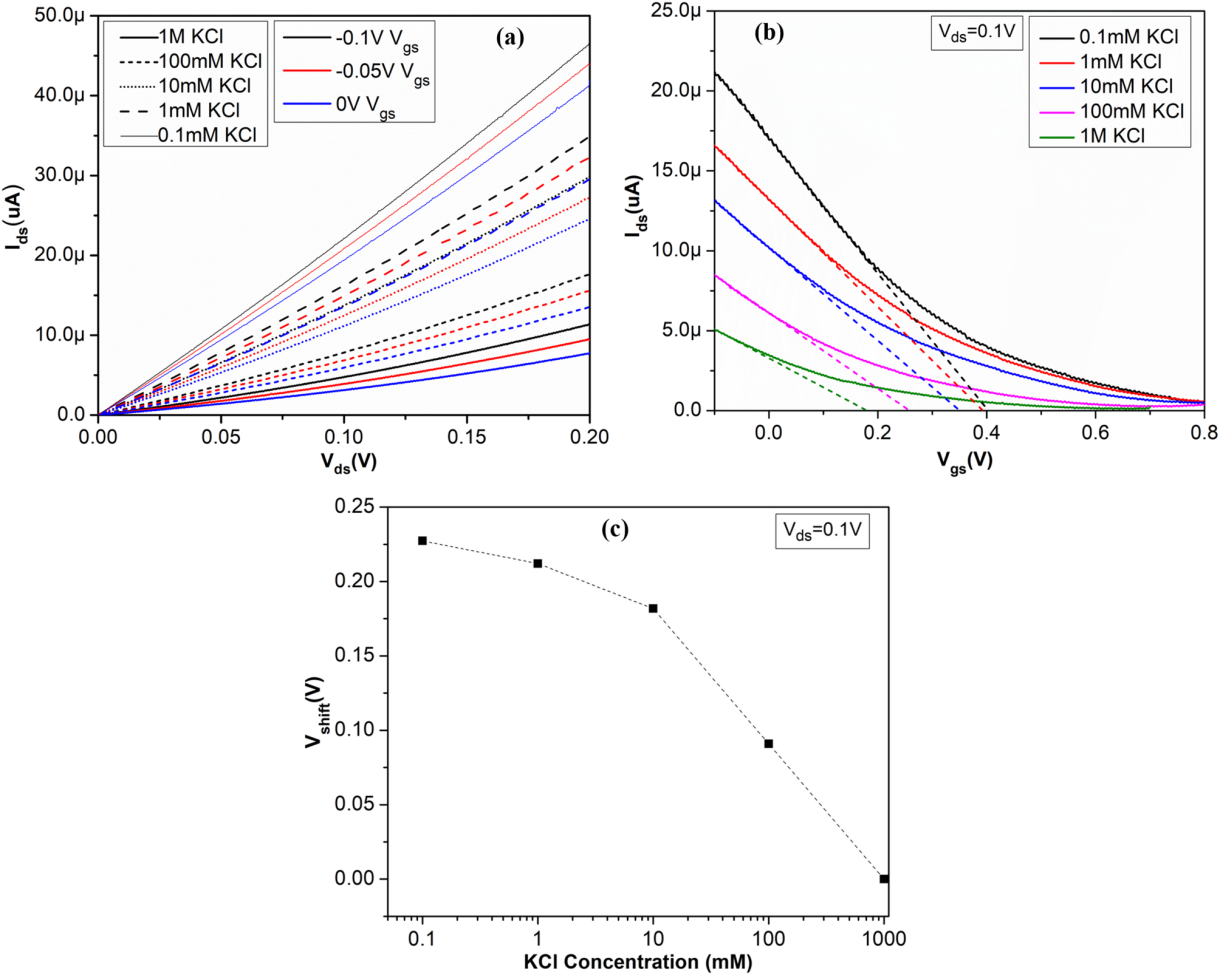
Electrical characteristics of a single device on the chip array (3 × 3) (**a**) Output characteristics for different applied gate voltages for different concentrations of KCl, (**b**) transfer characteristics for different concentrations of KCl at constant voltage V_ds_ ~ 0.1 V, and decrease in V_th_ for the increase in KCl concentrations are shown by fitting (dashed line) the linear region of the corresponding transfer curve for different concentrations and (**c**) V_shift_ for KCl concentrations is plotted while the value for 1 M KCl is taken as reference.

Figure 8a shows the output characteristics of the single device from the array (3 × 3 devices) for different KCl concentrations with liquid gate bias applied from − 0.1 to 0 V with 0.05 V incremental steps. In the transfer curve (Fig. 8b) the higher conductance of the device at more negative gate bias (V_gs_) indicates the p-type conduction in the s-SWNTs. The low current in off-state is due to m-SWNTs between the electrodes. The results shown in Fig. 8b reflect the significant modulation of conductance in the s-SWNTs network. The linear behaviour (due to diffusion of carriers) in output characteristics at low bias voltage shows that the overall resistances are dominated by significant contributions of SWNT-SWNT junctions in the random network than that of electrode metal-SWNTs contact junctions. This happens mainly due to the relatively larger channel length (~ 200 µm)^37^.

Transfer curve in the Fig. 8b shows that device turned off at V_gs_ ~ 0.8 V for 0.1 mM KCl concentration and this value decreases with increase in electrolytic concentration from 0.1 mM to 1 M. This reflects the dependence of conductance on ionic strength of the liquid. To determine sensitivity in electrolytic medium, there are different responsible mechanism, which includes majorly Schottky-barrier effect, electrolytic gating effect, capacitive modulation and mobility^38–40^. Device mobility is almost independent for the channel length of ≥ 2 µm^9^. As in our case, the channel length is ~ 200 µm; therefore, electrostatic gating will dominate over mobility effects. Here shifting of the transfer curve corresponding to different concentrations with a small change in the transconductance (slope of the linear region: dI_ds_/dV_gs_) shows the electrostatic gating effect^38^. In LG-CNTFET, the channel SWNTs adhere to the substrate surface, which is negatively charged under physiological conditions^41^. These negative charges are screened by the positive ions in electrical double layer (EDL) and positive charges (holes) in the SWNTs. Most of these negative charges are screened by the EDL because of its higher capacitance than the quantum capacitance of SWNTs. In other words, the SWNTs experienced reduced liquid gate potential by the potential drop across EDL in the solution.

For higher electrolyte concentration, the thickness of EDL reduces i.e. Debye screening length shortens; this results in higher EDL capacitance, which in-turns reduce the surface potential. Therefore, the more negative voltage is necessary to compensate for this change and the I_ds_-V_gs_ curve shifts towards more negative gate voltages^41^.

The drain current (I_ds_) in the linear region is given by the following relation^42^

1 $$I_{ds} = \frac{1}{2}\mu C_{eff} \frac{W}{L}\left[ {2(V_{gs} - V_{th} )V_{ds} - V_{ds}^{2} } \right]$$

where C_eff_ is the effective gate capacitance, which is mainly given by the in-series capacitances of the SWNTs/electrolyte (EDL capacitance) and the electrolyte/gate-electrode, µ is the carrier mobility, W and L are the gate width and length, respectively, V_gs_ and V_ds_ are the gate-source and drain-source voltages, respectively, and V_th_ is the threshold voltage. The transconductance g_m_ is expressed as:

2 $$g_{m} = \frac{{\partial I_{ds} }}{{\partial V_{gs} }} = \mu C_{eff} \frac{W}{L}V_{ds}$$

For extraction of V_th_, the extrapolation in the linear region (ELR) (also called as linear extraction (LE)) method was used^43,44^. The method is based on finding the gate-voltage axis intercept (i.e., I_ds_ = 0) of the linear extrapolation of the I_ds_–V_gs_ curve at its maximum first derivative (slope) point (i.e. the point of maximum transconductance (Fig. 8b)^45,46^.

For lowest KCl concentration (0.1 mM), the V_th_ value is highest (i.e. ~ 0.39 V) and as the concentration is increased to 1 M, the V_th_ is reduced to 0.17 V (Fig. 8b). To quantify the electrostatic gating effect, the shift in threshold voltage (V_shift_) vs KCl concentration for a single device of the array is plotted in Fig. 8c, where V_th_ of 1 M KCl is taken as reference for other KCl concentrations. As the KCl concentration decreases, the devices become more p-doped and correspondingly the increased V_shift_. The V_shift_ by 0.22 V towards more positive gate voltage, in a range of 5 decades of KCl concentration can be seen in Fig. 8c^14^. For the same LG-CNTFET device, the gate leakage current (I_gs_) of LG-CNTFET was measured for 0.1 mM to 1 M KCl concentration range under V_gs_ varying from − 0.4 to 0.8 V and constant V_ds_ ~ 0.1 V (see Fig. S1 in Supplementary Information). It was observed that the measured value of I_gs_ was in the range of ± 50 nA for all the KCl concentrations.

**Figure 9:**
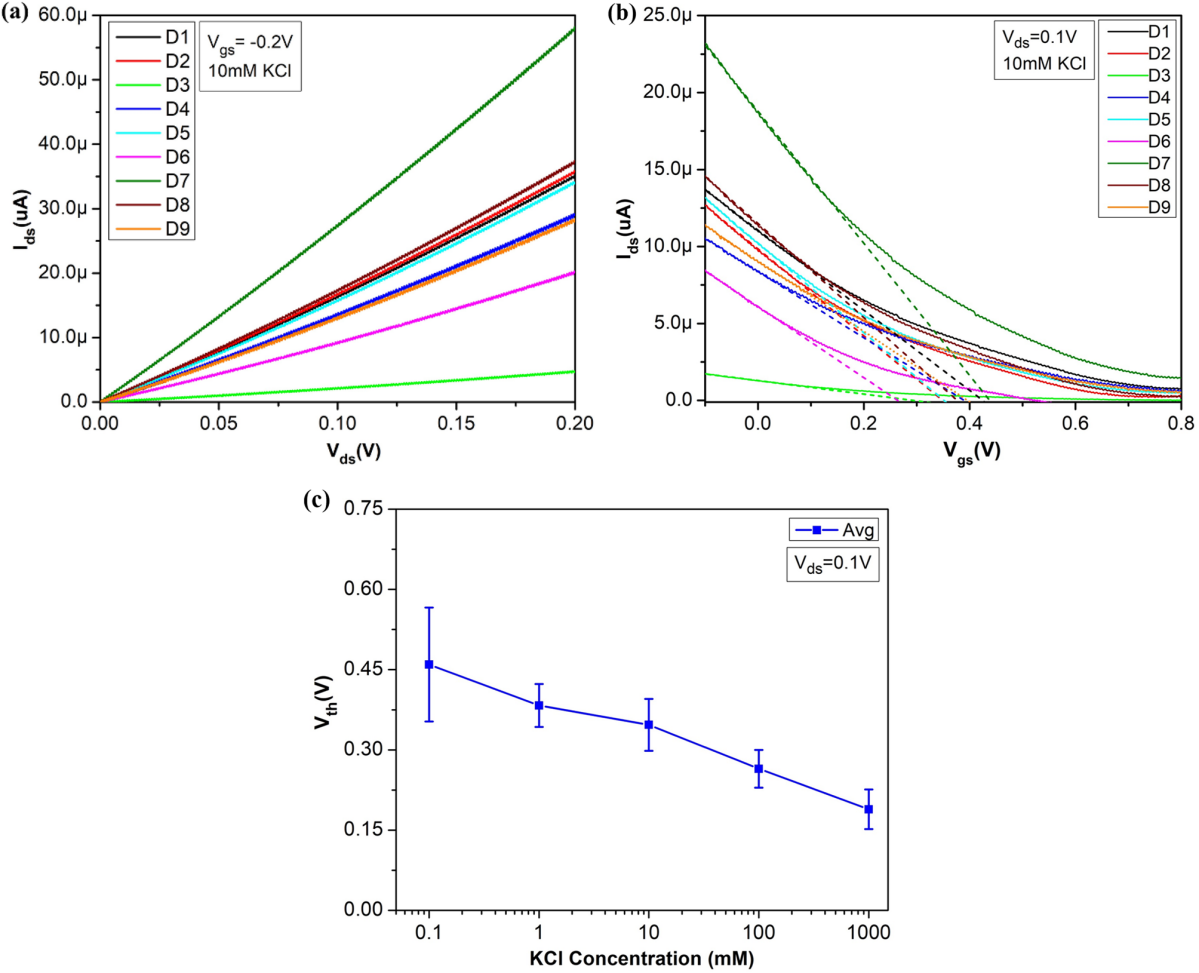
Electrical characteristics of 3 × 3 array of devices (**a**) Output characteristics for different devices at constant V_gs_ ~ − 0.2 V for 10 mM KCl concentration (**b**) transfer characteristics for different devices at constant voltage V_ds_ ~ 0.1 V and 10 mM KCl concentration, and similarity in V_th_ for different devices for fixed 10 mM KCl concentration is shown by fitting (dashed line) the linear region of the corresponding transfer curve and (c) plot of an average value of V_th_ for 9 devices of a chip for KCl concentrations along with error bar representation, while standard deviation as a source of error at each point.

The comparison of electrical measurements of multiple devices over a single chip (3 × 3 array) is essential to validate the uniformity among the devices. As explained in Fig. 5, the silicon shadow mask-based process results in good uniformity of source-drain resistances among the fabricated devices as well as chips covering the whole wafer area. In Fig. 9a, the output characteristics of all 9 devices of a chip (3 × 3 array) at a constant V_gs_ ~ − 0.2 V and 10 mM KCl concentration are plotted, where the characteristics of maximum devices from the array (7 out of 9) are similar except D3 and D7. The transfer curve as shown in Fig. 9b also confirms the uniformity among the devices. Using ELR method, the calculated V_th_ of 7 similar devices for 10 mM KCl concentration at constant V_ds_ ~ 0.1 V is 0.35 ± 0.06 V (Fig. 9b). The average value of threshold voltage for all 9 devices of a chip vs KCl concentrations is plotted in the form of an error bar by taking standard deviation as a source of error (Fig. 9c). As concentration increases, the average threshold voltage also shows a linear decrease for the array of devices, similar to the case of the individual device as explained for Fig. 8c. For these 3 × 3 array of LG-CNTFET devices, measured I_gs_ was within ± 50 nA for 0.1 mM to 1 M KCl concentrations under constant V_ds_ ~ 0.1 V for varying V_gs_ from − 0.4 to 0.8 V (see Fig. S2 in Supplementary Information).

Conventionally, the fabrication process of LG-CNTFETs involves thin film preparation of CNTs over a large size substrate/wafer, followed by their patterning using photolithography and removal/etching of CNTs from the undesired area using oxygen plasma^23,47–50^.

In Table 1, we compare our shadow mask-based s-SWNTs patterning approach with the reported fabrication methods for the realization of similar LG-CNTFET array of devices^49,51–55^. In most of the cases, for such semiconductor device array fabrication, the use of mixture (s-/m-) SWNTs results in variation in device-to-device characteristics and degradation of device performance especially due to m-SWNTs. In our fabricated devices, s-SWNTs were used for the LG-CNTFET fabrication, which results in uniform electrical performance of devices in an array (Fig. 9). The competitive advantage of our developed shadow-mask based chemical-free process for the fabrication of LG-CNTFET is the minimization of the intermediate steps namely photolithography, etching and photoresist removal, which makes the device manufacturing simpler and economic.

**Table 1:** Comparison of technologies for the fabrication of LG-CNTFET array.

Deposition of SWNTs		Patterning of SWNTs			Realization of LG-CNTFET array			References
Method	Type of SWNTs (s & m)	Technique	Resist-based/chemical-free	Size of substrate	Fabrication steps	Passivation layer used	Total number of processes	
Spin-coating	s-SWNTs	Photolithography of spin-cast SWNTs	Resist-based	100 mm diameter	Spin-coating of SWNTs → Photolithography for source-drain electrodes fabrication → Metal (Ti) deposition followed by lift-off → Photolithography for bond pads fabrication → Metal (Au) deposition followed by lift-off → Photolithography for protection of SWNTs between the electrodes → Etching of SWNTs from the regions outside the electrode gap	Not mentioned	07	^51^
	m-SWNTs							
Drop-casting	Mixture (s-/m-) SWNTs	Dielectrophoresis	–	6.4 × 3.0 mm^2^	Metal (Pt) deposition → Photolithography for source-drain electrodes fabrication → Deposition of passivation layer → Photolithography for electrodes area and contact pads opening → Drop casting of SWNTs → Dielectrophoresis	Silicon oxide/silicon nitride	06	^52^
Drop-casting	Mixture (s-/m-) SWNTs	Dielectrophoresis	–	3.2 × 3.2 mm^2^	Metal (WTi & Pt) deposition → Photolithography for connector leads fabrication → Deposition of first^a^ passivation layer → Photolithography for contact pads opening → Metal (Pt) deposition → Photolithography for electrodes fabrication → Drop casting of SWNTs → Dielectrophoresis → Photolithography for second metal (Pt) layer → Metal (Pt) deposition followed by lift-off → Deposition of second^b^ passivation layer	^a^Silicon oxide/silicon nitride and ^b^SU8	11	^53^
CVD	Mixture (s-/m-) SWNTs	Photolithography of catalyst layer followed by SWNTs growth	Resist-based	Not mentioned	Deposition of catalyst (Co) layer → Photolithography for patterning of catalyst (Co) → CVD-based growth of SWNTs → Photolithography for source-drain electrodes fabrication → Metal (Ti/Au) deposition followed by lift-off	Not mentioned	05	^54^
CVD	Mixture (s-/m-) SWNTs	Photolithography of grown SWNTs	Resist-based	10 × 12 mm^2^	Deposition of catalyst (Fe) layer → CVD-based growth of SWNTs → Photolithography to create alignment marks (lift-off) → Etching of SWNTs from the regions where metal to be deposited → Metal (Ti) deposition followed by lift-off for alignment marks creation → Photolithography for patterning SWNTs between the electrodes → Etching of SWNTs from the regions outside the electrode gap → Photolithography for source-drain electrodes fabrication → Metal (Ti/Au) deposition followed by lift-off → Deposition of passivation layer → Photolithography for contact pads opening	Aluminium oxide	11	^49,55^
Spray-coating	s-SWNTs	Shadow mask technology	Chemical-free	76.2 mm diameter	Metal (Cr/Au) deposition → Photolithography for source-drain electrodes fabrication → Deposition of passivation layer → Photolithography for electrodes area and contact pads opening → Spray coating of s-SWNTs using silicon shadow mask	Silicon oxide/silicon nitride	05	Our work

*Ti* titanium, *Au* gold, *Pt* platinum, *W* tungsten, *Co* cobalt, *Fe* iron, *Cr* chromium.

## Conclusions

In conclusion, chemical-free shadow mask lithography along with spray coating has been successfully used for patterning the s-SWNTs to fabricate a uniform array of LG-CNTFET devices. For demonstration, the array of LG-CNTFET platform devices was electrically tested with 0.1 mM to 1 M KCl concentrations using Ag/AgCl gate electrode, immersed in fabricated PDMS well. The applied V_ds_ and V_gs_ voltage ranges were 0–0.2 V and − 0.4 to 0.8 V, respectively for acquiring output and transfer characteristics of the device array. Majority of devices in a single chip have resistances in the range of 1–3 kΩ, which results in their uniform electrical performance. For example, V_th_ of similar 7 devices for 10 mM KCl concentration at constant V_ds_ ~ 0.1 V is 0.35 ± 0.06 V. The average value of measured V_th_ for complete device array decreases from 0.46 to 0.19 V with increased KCl concentration from 0.1 mM to 1 M. The developed process is contamination-free, rapid and would be economical to fabricate a uniform array of s-SWNTs-based highly sensitive LG-CNTFET devices for a variety of applications in biochemical sensors.

## Supplementary Information

Supplementary Information
