# Supplementary material for: Chemical-free and scalable process for the fabrication of a uniform array of liquid-gated CNTFET, evaluated by KCl electrolyte
Source: Sci Rep. 2021 Feb 17;11:3979. doi: 10.1038/s41598-021-83451-2 (PMC7889891; doi:10.1038/s41598-021-83451-2)
Supplement: Supplementary file 1 — Supplementary Information [file 41598_2021_83451_MOESM1_ESM.docx]

**Supplementry Information**

Chemical-free and scalable process for the fabrication of a uniform array of liquid gated CNTFET, evaluated by KCl electrolyte

Pankaj B. Agarwal,*^,†,‡^ Navneet Kumar Thakur,^†^ Rishi Sharma,^†,‡^ Parul Singh,^†^ Joshy Joseph, ^⊥,‡^, and Chaturvedula Tripura ^#,‡^

^†^ Smart Sensors Area, CSIR-Central Electronics Engineering Research Institute (CSIR-CEERI), Pilani, Rajasthan, 333031, India

^‡^ Academy for Scientific and Innovative Research (AcSIR), Ghaziabad, Uttar Pradesh, 201002, India

^⊥^ Chemical Sciences and Technology Division, CSIR-National Institute for Interdisciplinary Science and Technology (CSIR-NIIST), Thiruvananthapuram 695019, India

^#^CCMB-Annexe-II, Medical Biotechnology Complex, CSIR-Centre for Cellular and Molecular Biology (CSIR-CCMB), Uppal Road, Uppal, Hyderabad 500039, Telangana, India

*Corresponding author, Email: agarwalbpankaj@gmail.com

**KEYWORDS:** Chemical-free lithography, Silicon shadow mask, Carbon nanotubes, CNTFET, Chemical and biosensors.

**Gate leakge current measurements of fabricated LG-CNTFET devices**

| **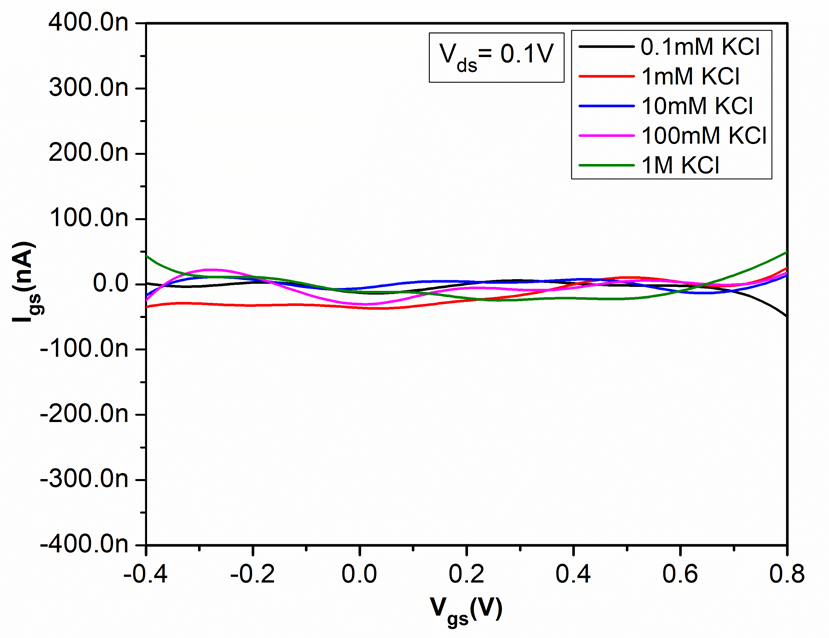**  **Figure S1**. Gate leakage current of a single device on the chip array (3x3) for different applied gate voltages with different concentrations of KCl at constant voltage V_ds_~0.1 V. |
| --- |
| **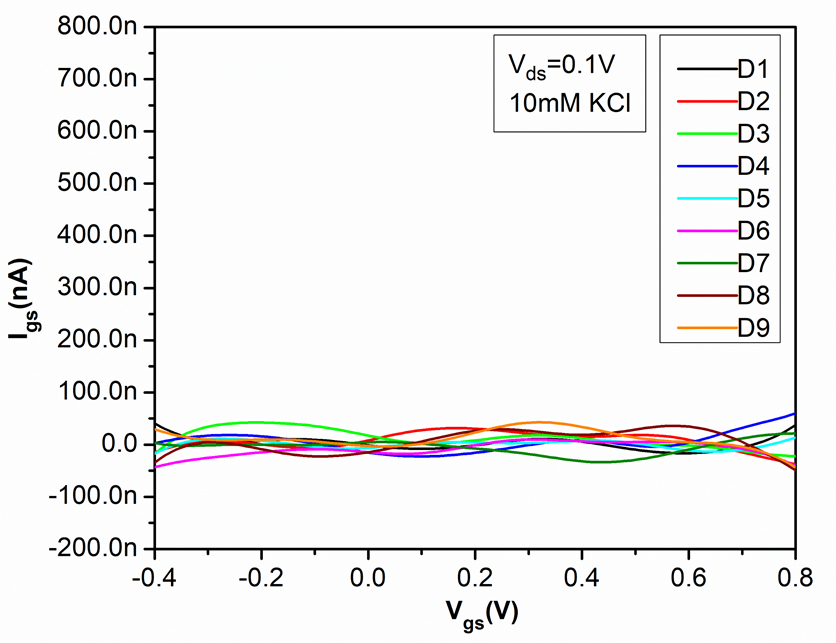**  **Figure S2.** Gate leakage current measurements of 3 X 3 array of devices (D1-D9) at constant voltage V_ds_~ 0.1 V and 10 mM KCl concentration. |
